# An automatic method for assessing structural importance of amino acid positions

**DOI:** 10.1186/1472-6807-9-10

**Published:** 2009-03-04

**Authors:** Michael I Sadowski, David T Jones

**Affiliations:** 1 Computer Science Department, University College London, Gower St, London, WC1E 6BT, UK; 2 Division of Mathematical Biology, National Institute for Medical Research, The Ridgeway, Mill Hill, London, NW7 1AA, UK

## Abstract

**Background:**

A great deal is known about the qualitative aspects of the sequence-structure relationship, for example that buried residues are usually more conserved between structurally similar homologues, but no attempts have been made to quantitate the relationship between evolutionary conservation at a sequence position and change to global tertiary structure. In this paper we demonstrate that the Spearman correlation between sequence and structural change is suitable for this purpose.

**Results:**

Buried residues, bends, cysteines, prolines and leucines were significantly more likely to occupy positions highly correlated with structural change than expected by chance. Some buried residues were found to be less informative than expected, particularly residues involved in active sites and the binding of small molecules.

**Conclusion:**

The correlation-based method generates predictions of structural importance for superfamily positions which agree well with previous results of manual analyses, and may be of use in automated residue annotation piplines. A PERL script which implements the method is provided.

## Background

Over the course of evolutionary time proteins which retain a particular molecular function accumulate neutral mutations to their sequences; these mutations in turn generate functionally neutral changes to the structure of the protein [1].

The location of an amino acid residue in the sequence and structure constrains its mutability according to how easily the mutation can be accommodated without disruption [2,3]. Thus buried positions generally accept mutations less readily than those exposed to solvent and where function is conserved it is extremely rare to find mutations to active site residues [4,5].

These broad details of the relationship between protein sequence and structure are well established but more detailed patterns are difficult to assess on a global scale and it becomes necessary to examine superfamilies individually to determine which positions may be most important for maintaining structure and function.

A large number of manual analyses have been published [e.g. [6-10]] which identify structurally important residues in particular protein families, however these are time-consuming, rely on extensive knowledge of the details of the family in question and may be subjective. A quantitative assessment of the importance of particular amino acid sites in a family of proteins by an automatic method would be an important step towards standardising such assessments.

Such a method would also be useful as an input for incorporation into analysis pipelines such as those used to annotate functional residues or predict the mutational consequences of SNPs [e.g. [11,12]].

In this paper we present a quantitative examination of the structural importance of amino acid positions in six superfamilies taken from the CATH database [13] using a modified version of a method originally developed for predicting functionally important residues [14]. This method simply compares global structural change with the degree of mutational difference at a given position in a multiple alignment and corrects for the overall level of mutation as a measure of the degree of mutational constraint experienced by that position as a consequence of its importance for the structure.

The results are found to agree with known principles of protein sequence-structure relationships in general and in a specific case-study of the globin superfamily. We further show that similarity scores using subsets of structurally important residues are highly correlated with structural similarities of distantly related proteins. We demonstrate that the results are generally robust to changes in the definitions of the categories and the measures of residue similarity and structural divergence, indicating that they are not critically dependent on the details of these definitions.

We conclude that this method produces a meaningful ranking of the importance of amino acid residues for protein structure and is a potentially useful addition to protein residue annotation pipelines. A PERL script which implements the method is provided [see additional file 1].

## Results

The method aims to determine which sequence positions in a superfamily are most strongly indicative of global structural change by finding the correlation between mutational changes at a given position with a global RMSD score.

To assess the performance of this method it was run on multiple alignments of six CATH superfamilies containing a range of closely and distantly related protein pairs (listed in Table 1) and tested for its relationship with previously studied structural features.

**Table 1:** Dataset composition

***Superfamily***	***CATH code***	***Min ID***	***Median***	***Max***	***N***
**Amylase**	3.20.20.80	6%	11%	74%	40
**Cupredoxin**	2.60.40.420	9%	20%	90%	35
**Globins**	1.10.490.10	4%	19%	89%	71
**Jellyroll/Capsid**	2.60.120.20	4%	11%	89%	53
**Lysozyme C**	1.10.530.10	5%	34%	87%	17
**PLA**_**2**_	1.20.90.10	5%	40%	90%	44

Superfamily name, CATH code, %ID ranges and dataset sizes are shown

### The Distribution of Site-Specific Sequence/Structure Correlations Varies By Superfamily

**Figure 1:**
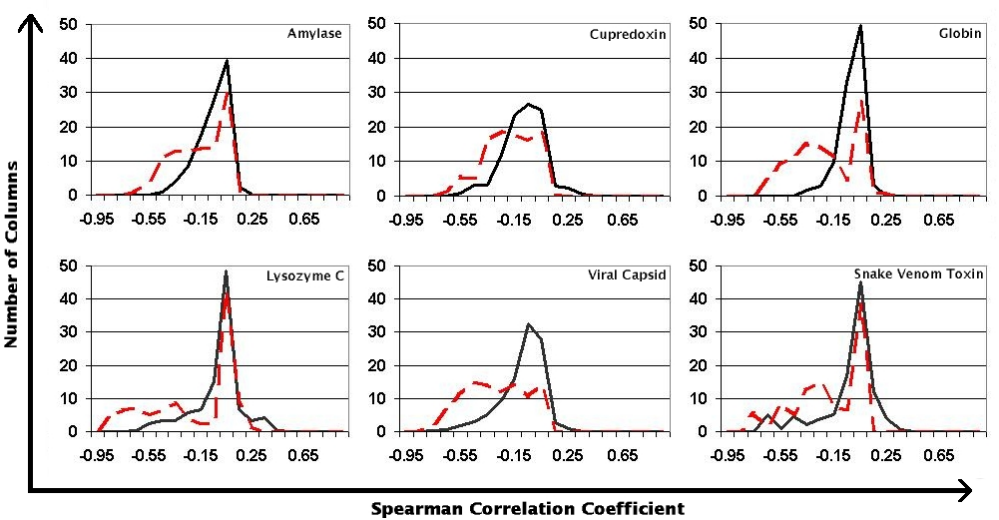
**Distributions of positional rank-correlations with global structural change for six superfamilies**. Histograms of Spearman's rank correlation coefficients with bin widths of 0.05 for the six datasets (dashed black lines). Partial correlations controlled for global sequence-structure similarity are also plotted (solid red lines). Datasets are ordered left-to-right: amylase, cupredoxin, globin, lysozyme, viral capsid (jellyroll), phospholipase A_2 _(snake venom toxin).

Figure 1 depicts the distribution of rank-correlation coefficients for each of the six superfamilies (red, dashed lines). In order to account for the overall sequence/structure relationship the distributions of partial correlations which account for the relationship to global sequence similarity are also shown (black lines). We use the partial correlation measure in later analyses since it removes the background noise from sequence similarity.

Although the raw correlations at the majority of positions are highly significant the partial correlations for most of the superfamilies show strong peaks around zero, suggesting that the majority of positions in these alignments provide information about structural similarity only as far as they contribute to the overall sequence similarity.

However, all six families also contain positions with partial correlations of -0.3 or better, which are highly significant given the large number of pairs in each dataset. The proportion of strongly correlated positions varies from 2% for the globins to 13% for the snake venom toxins. The most strongly correlated sequence position is found in the viral capsids and has a partial correlation value of just over -0.7. This residue is highly conserved within each of the three major groups of viral coat protein domains in this dataset, but the residue found in each group differs in each case.

### Structurally Informative Positions Reproduce Previous Structural Observations

We examined the composition of the multiple alignment columns with respect to their degree of correlation with structural change. In order to determine whether the structural correlations were meaningful we examined the relationship between amino-acid composition, secondary structure and structural position with the correlation measure using chi-squared tests (see methods for category definitions).

The column containing each residue was assigned to one of three categories: significantly correlated, significantly anticorrelated and uncorrelated according to the consensus of four partial correlation coefficients (derived using two RMSD measures and two different score matrices, see methods). To aid clarity we refer to these classes as *Structurally Informative*, *Structurally Uninformative *and *Structurally Neutral *respectively.

The results of chi-square tests are summarized for the three categories in tables 2, 3 and 4. In order to ensure that the results were not due to a particular dataset we removed each set in turn and repeated the analysis to see how the results would vary.

**Table 2:** Associations between structural correlations and residue types

**Type**	**Chi-sq**.	**Significance**	**N**	Category
**Functional**	602.4	< 4e-15	3201	**Uninformative**

**Intermediate**	24.6	< 1e-04	5220	**Neutral**

**Interface**	180.52	< 4e-15	2677	**Neutral**

**Buried**	261.5	< 4e-15	7033	**Informative**

**Exposed**	398.1	< 4e-15	10547	**Neutral**

Chi-squared values (Chi-sq) with significance levels are shown for each of the five residue types: Buried, intermediate, exposed, functional and interface. See text for definitions. Significance values are corrected for five tests using Bonferroni's correction. The "category" column indicates the direction of correlation overrepresented for that class where probabilities were significant at the 5% level.

**Table 3:** Associations between structural correlations and secondary structure types

**SS**	**Chi-sq**.	**Significance**	**N**	Category
**Bend**	27.7	< 1e-05	2140	**Informative**

**Alpha Helix**	166.1	< 1e-14	13021	**Uninformative**

**Turn**	26.3	< 2e-05	3527	**Neutral**

**Coil**	15.0	< 4e-03	5056	**Neutral**

**3/10 Helix**	75.1	< 1e-14	1568	**Uninformative**

**Bridge**	2.2	> 0.05	406	**Not Significant**

**Strand**	114.6	< 1e-14	2960	**Neutral**

Chi-squared values with significance levels are shown for each of seven secondary structure (SS) types as defined by DSSP (no structure in the dataset contained a pi-helix). Significance values are corrected for seven tests using Bonferroni's correction. The "category" column indicates the direction of correlation overrepresented for that class where probabilities were significant at the 5% level.

**Table 4:** Associations between structural correlations and residue types

**AA**	**Chi-sq**.	**Significance**	**N**	Category
**A**	36.7	< 4e-15	3339	**Uninformative**

**C**	137.6	< 4e-15	982	**Informative**

**D**	48.5	< 5e-10	2373	**Neutral**

**E**	58.3	< 4e-12	1934	**Uninformative**

**F**	93	< 4e-15	1621	**Uninformative**

**G**	33.9	< 4e-15	2830	**Informative**

**H**	7.4	> 0.05	1098	**Not Significant**

**I**	8.4	> 0.05	1729	**Not Significant**

**K**	29.1	< 1e-05	2369	**Uninformative**

**L**	52.8	< 1e-10	2812	**Informative**

**M**	9	> 0.05	762	**Not significant**

**N**	30	< 1e-05	1963	**Uninformative**

**P**	49.9	< 1e-10	1540	**Informative**

**Q**	12.9	< 0.025	1181	**Uninformative**

**R**	37.5	< 2e-07	1419	**Uninformative**

**S**	11.6	> 0.05	2172	**Not Significant**

**T**	0.6	> 0.05	1926	**Not significant**

**V**	21.1	< 1e-03	2441	**Neutral**

**W**	38.8	< 1e-07	745	**Informative**

**Y**	17.3	< 0.005	1625	**Informative**

Chi-squared (chi-sq) values with significance levels for structural correlations (see Methods) are shown for each of the twenty amino-acid types. Significance values are corrected for twenty tests using Bonferroni's correction. The "category" column indicates the direction of correlation overrepresented for that class where probabilities were significant at the 5% level.

Buried core residues were found to be in structurally informative columns more often than expected by chance, exposed residues were more likely to be neutral and functional residues were more likely to be uninformative. These results were unaffected by removal of any single dataset or the change of criteria of classification (see below).

Intermediately exposed residues were significantly more likely to be informative than expected from a random distribution of residues, however this effect disappeared with the removal of either the globin or snake venom toxin datasets.

Interface residues were significantly more likely to be informative than expected, however this effect was exclusively due to the viral capsid proteins, which contained a substantially greater number of interface residues than the other superfamilies. Once these were removed the interface residues for the remainder were significantly more likely to be uninformative.

For the secondary structure classes, four results were consistently unchanged by removing each dataset: bend residues were always significantly overrepresented in informative columns; 3–10 helix residues were always significantly overrepresented in uninformative columns and coil and strand residues were always significantly overrepresented in neutral columns. Alpha-helical residues were more likely to be uninformative than expected, however this was solely due to the globin dataset, removal of which put these residues in the neutral class. Turn regions were found to be neutral except when the PLA_2 _sequences were removed, which moved them to the informative class. Isolated beta-bridge residues showed no consistent preference.

The sequence composition results similarly make sense in light of previous structural analyses. Cysteine, leucine and proline were consistently overrepresented in informative columns; tryptophan, tyrosine and glycine were similarly overrepresented in most cases. Arginine and aspartic acid were consistently overrepresented in structurally neutral columns; valine in the majority of cases. Phenylalanine was consistently overrepresented in uninformative columns, with asparagine and glutamic acid in 5/6 cases.

Overall the patterns agree with expectations based on earlier studies [1-5] although the observations relating to functional residues and secondary structures have not to our knowledge been previously described.

Of course it is always difficult to categorize data deriving from continuous scales in a consistently meaningful way and this may introduce some bias. To address this issue we varied the thresholds for the solvent accessibility based patterns and functional residues and found that although exposed and intermediate residues varied in their tendency to be neutral or uninformative the main results did not change significantly. Incorporating more residues surrounding non-protein atoms in the PDB files in the functional class also did not change the findings presented until a significant fraction of the sequence was included.

To further validate the method with a detailed case-study we used the globin superfamily, which has been the subject of considerable study, as a test case.

### Structurally Informative Positions in the Globins

**Figure 2:**
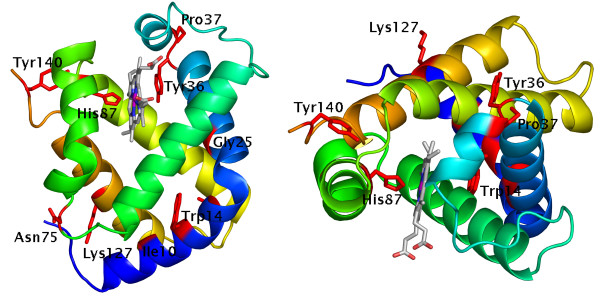
**Significantly selected positions in chicken haemoglobin**. The structure of chicken haemoglobin (PDB:1hbr) chain A is shown; residue backbones are coloured blue to orange from N to C terminal. Residues most strongly correlated with structural change are depicted in red. Figure created with PyMol (Delano Scientific, San Carlos, CA, USA).

Figure 2 depicts the top 10 positions selected in the globin family mapped on the structure of chicken haemoglobin [PDB:1hbr chain A; [15]].

**Gly 25 **is known to be very highly-conserved in the globin family owing to its location where two helices cross [8]. In 22 of the 70 sequences this residue has mutated. Many of these changes are conservative (e.g. G->A, S) however there are other classes of globin such as the truncated globins (e.g. 1kr7) in which more extreme changes are found (e.g. G->V); in such cases the closest approach of the two helices is greater by 0.5 Å or more. In all structures with a glycine at this position this is annotated by our method as a functional residue, however in every case where the residue has mutated its class has changed.

**His 87 **is involved in chelating the haem group. This position varies in only three cases: 1dly, 1mwb and 1ew6 which are from green algae, a cyanobacterium and a polychaete marine worm respectively and differ functionally from vertebrate globins, which are the majority of this set. Inspection of the alignment shows that the functionally equivalent histidines are at slightly different sequence positions in these examples. In these cases this residue is classed as exposed by the automatic annotation described above.

**Ala 17 **packs into the core from helix A (blue in the figure). This position is generally hydrophobic with its identity conserved only for close homologues. This suggests that it is useful in distinguishing relatively closely-related sequences from more distant ones.

**Ile 10 **extends from the centre of helix A into the core at the point where helices A, D and H meet. In vertebrate globins this is mostly valine but in others where the N-terminus is truncated it is generally a more polar site (e.g. serine), hence it may act as an indicator of the length of the N-terminus.

**Tyr 140 **is the penultimate residue in 1hbr and forms a side-chain/main-chain hydrogen bond with the oxygen of V93 in the loop preceding helix F. A similar interaction is conserved within groups (e.g. 1cqx, 1vhb, 1gvh) which conserve a particular residue (i.e. Tyr in most globins, Glu in 1cqx etc.) but with different details of interaction: where a tyrosine appears at this position we find the interaction to be a side-chain/main-chain hydrogen bond; in 1cqx etc. this is a side-chain/side-chain bond to more than one partner. This residue is absent in the truncated globins. The side-chain also forms part of the interface in 29 of the family members considered here. A tyrosine at this position is found to be a necessary, but not sufficient, condition for an involvement in an inter-chain interface.

**Tyr 36 **is a surface residue. It is not immediately obvious how this position is constrained by structure however its role in 1hbr may be suggestive: it is involved in a sidechain-sidechain hydrogen bond with glutamine Q103 in the penultimate helix. This non-local contact may be involved in formation of the final tertiary structure. The nature of this interaction differs slightly in other structures examined. For example, in the 101 m sperm whale myoglobin structure there is now an aromatic interaction between H36 and F106 with an additional hydrogen-bond to E109. In the truncated globin 1kr7 H18 packs against N81 although it is not apparently hydrogen-bonded. A local aromatic interaction with Y21 now occurs and this tyrosine hydrogen-bonds to N81. In 1tu9, another truncated globin structure, this position is now a serine, S32, which locally hydrogen bonds with Q34. In the marine worm globin 1ew6 this has become a hydrophobic interaction with the tyrosine at this position (Y28) now sandwiched between two lysines: K27 and K99. Within another closely-related group (1g0b, 1g08 *et al*.) this has become a Phe and now interacts with Leu100.

**Lys 127 **is highly conserved across a great evolutionary distance. Again a surface residue, this is also involved in a sidechain-sidechain hydrogen bond, this time with an aspartic acid at position 6, uniting the C- and N-termini. Where this residue has mutated this interaction is not conserved. In 1hbr this residue is an interface residue. Although this role is not completely conserved it is found in only one case where this residue is not lysine (1ith).

**Pro 37 **is very strongly conserved across the majority of the superfamily (D or Q in 6 of 70 sequences) and is adjacent to the lysine involved in a non-local sidechain hydrogen bond as found at position 36. As observed above the non-local interaction at this site is very highly conserved and where the proline has mutated this interaction seems to have altered concomitantly.

**Trp 14 **is a strongly-conserved aromatic residue, predominantly tryptophan. This is involved in packing between helices A/D and forms a "ridge" which fits into the "groove" made by L63, G67 and V70. Mutations to other residue types at this position are associated with different N-terminal conformations.

**Asn 75 **is highly solvent-accessible and conserved mostly over short evolutionary distances. In some cases the sidechain of the residue at this position is involved in a local hydrogen-bonding interaction. This residue is found to be exposed in nearly all family members.

Several core positions were anticorrelated, that is they were found to contribute more noise than signal to the level of sequence-structure correlation derived using them. In 1hbr these are positions 32, 105 and 109. Each of these has a mean of < 5% accessibility across the entire set of sequences. This may seem surprising as it is expected that core residues will be more greatly conserved, however two complementary explanations can be suggested.

In the first place these residues are consistently located in the hydrophobic core and therefore must conserve their hydrophobicity. Since there are several possible residues which are equally acceptable (e.g. I, V, L) these positions are candidates for multiple substitutions. In the second place their positioning in contact with other sidechain residues provides the maximum possible repacking in response to changes in shape or volume. Thus the structural effect on the backbone position is likely to be minimal.

### Conservation of Structurally Informative Residues Can Improve Predictions of Structural Similarity for Distant Homologues

As a final test of this method we examined whether the conservation of the more highly correlated residues was more indicative of structural similarity than that of the least highly correlated residues. Although we expect this to be the case it is possible that the combination of several residues might not increase the amount of information since they may be correlated or anticorrelated with one another.

**Figure 3:**
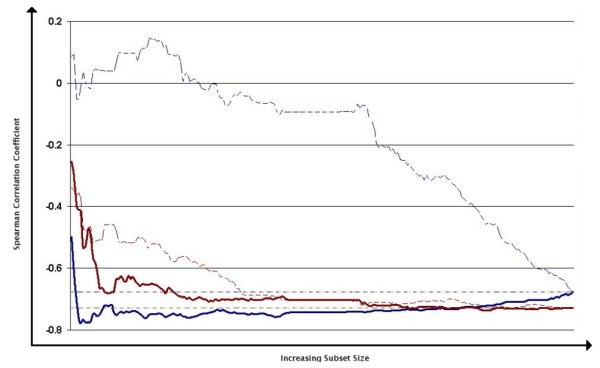
**Improving sequence-structure correlations with strongly correlated positions: example with the globin superfamily**. Spearman's rank correlations between sequence similarity scores calculated from subsets of alignment positions with TM-align RMSD scores are plotted for increasing subset size from left to right. Y-axis: correlation coefficient. Data are separated into close (> 30% identical) homologues (red lines) and distant homologues (blue lines). Thick lines depict the effect of adding positions in order of correlation from most to least; thin dashed lines show the effect of adding positions in reverse order. Dashed horizontal lines show correlations for full sequence identities for the distantly (upper line at -0.68) and closely (lower line at -0.73) related sets.

Figure 3 shows the rank correlations of sequence identity scores generated from subsets of increasing size with TM-align RMSD scores, using the globin sequences as an example. The data are split into close homologues (≥ 30% ID) and distant homologues (< 30%ID). For comparison the results of adding positions in reverse order are also plotted.

For distantly related pairs the correlation based ranking is clearly effective, with a minimum correlation of -0.778 compared to -0.676 for the full sequence similarity score. Using the Fisher transformation this difference has a Z-score of 23 and accounts for ~14% more of the variance in the relationship. This is achieved using only the top five positions. Scoring with the ranks reversed shows that the positions at the bottom of the list are indeed more noisy than would be expected and cumulatively push the overall correlation above zero. The steep correction before returning to the full sequence correlation score again shows that the highly correlated residues contribute significant information.

By contrast the closely-related sequence pairs do not at any point exceed the level of correlation attained by the full sequence similarity scores but instead gradually approach the correlation of the full ID score from above. For both the forward and the reversed rankings there is an initial steep drop as sufficient information to make a reasonable classification is reached (about 20 residues), however after this the correlation of the forward ranked set continues to drop whereas the reverse rankings plateau very early.

## Discussion

In this paper we present an automatic method for assigning relative structural importance to sequence positions and shown that the results accord well with prior expectations. The main results are summarised and discussed below.

### Most Buried Positions are Better Correlated with Structural Change Than Sequence Change

Buried core positions are significantly correlated with structure, over the level expected based on overall sequence-structure correlation. Similarly, exposed positions are overall equally correlated with structure as would be expected from the relationship with overall sequence change. Given that buried positions experience mutational constraints as a result of their structural location this makes sense and agrees with previous results. Exposed positions are similarly explicable. We also find that some buried positions are apparently able to mutate without affecting the structure, which is somewhat more surprising. One possible explanation is for a position to be in contact with sidechains with sufficient degrees of freedom to adopt a different packing without altering the backbone conformation.

The overall neutrality of interface residues outside the viral capsid proteins was surprising to us as many of the more detailed analyses suggested that these positions were frequently strongly correlated. However given that these are essentially exposed positions which have acquired a functional constraint they could be regarded as a more slowly-evolving class of exposed residue, therefore providing information about structural change in concert with overall sequence changes. Residues which expose an intermediate level of surface area to the solvent are similarly correlated with sequence and structural change to the same extent, which accords with the existence of an intermediate level of mutational constraint due to structure.

Residues in contact with non-protein atoms in the structures, which can be regarded as functional residues, are found to be poorly correlated with structural change. Once the relationship with sequence change is accounted for these positions actually provide less information on structural changes than would be expected. Varying the threshold for the contact distance to be more permissive only strengthened the relationship until the point at which a large proportion of the sequence was being included. This shows that the relatively smaller number of these residues compared to the other types was not the cause of this result.

The reason for this is simply that on the whole these residues are much more correlated than the majority of columns. Consequently there is a much greater range of structural similarities given conservation of a functional residue than for many more variable positions.

In any case, since the main selection pressure in these groups is to maintain function any structural changes we observe (assuming function has not changed) must be neutral as far as functional change is concerned; therefore conservation of such sites could be expected to be uninformative. The interesting conclusion is that while functional residues are crucial for distinguishing correct from incorrect (super)family assignments they are detrimental in determining which of a set of sequences is likely to be the most similar structurally.

### Bends correlated, 3/10-Helices anticorrelated, Coils, Strands uncorrelated

We find that elements in bends contain more information about structural change than would be expected from their correlation with sequence similarity. Conversely, mutations to residues in 3–10 helices are significantly likely to correlate poorly with structural change given their correlation with sequence change. Turns, coils, helices and strands are more likely to be uncorrelated than expected.

Overall this suggests that most secondary structure types do not bias the importance of residues for the structure compared to the significant effect of solvent accessibility. Since a residue in a secondary structure can be buried or exposed this is hardly surprising. Bends are perhaps explicable in terms of torsional restraints on compatible residues: if a bend is conserved from one protein to another it is likely to be necessary to maintain particular residues compatible with the local curvature; where a bend and a non-bend are aligned a mutation may well indicate the loss of this local pattern. 3–10 helices are unusual but make up ~5% of the residues in our dataset.

### Bulky Hydrophobic residues, Cysteines, Prolines & Glycines are Informative

Analysis of correlation patterns with reference to sequence composition finds cysteine to be the most significantly likely to exist in a column with more information on structural change than expected. Since many of the proteins in this study contain disulphide bonds in varying patters this is another expected result. Proline and glycine are similarly overrepresented (although glycine inconsistently so), which given their torsional properties is also as expected.

In addition to these cases two other residues are significantly informative: leucine, and tryptophan. Given their hydrophobicity this is likely to be associated with their tendency to be buried; additionally the size of tryptophan is likely to be a factor since mutation to or from these in a buried position is likely to entail repacking to accommodate the volumetric change.

Residues which tend to be exposed are likely to be less informative, which accords with the observations above, however we also found F and V to be less well correlated than expected.

### Sequence Identity Scores Using Subsets of Structurally Informative Positions can Improve Prediction of Structural Similarity

Generating restricted sequence identity scores for subsets of aligned positions using this method would in principle allow the range over which structural similarity can be reliably predicted to be increased: at present for most superfamilies once < 20% ID is reached the most similar structures cannot be distinguished from the least similar since for very distantly related proteins the majority of positions have become mutationally saturated and therefore contribute only chance similarities.

Figure 3 shows that conservation of the positions most highly ranked by this method remains informative within this range, although naturally the limited information available adds noise to the comparison between more similar sequences.

It is interesting that although the more highly correlated residues are clearly useful for closely homologous proteins the best correlated score in the globin superfamily was only reached with the full sequence identity. This suggests that for closely related pairs we are simply removing information by ignoring certain positions. Alternatively it may be the result of the sensitivity of the correlation coefficient to outliers in the data: if one structure in a set is very different from the remainder this method will assign high correlations to positions which discriminate the highly divergent structure from the remainder.

To address this it would be better to separate the dataset into closely related pairs and distantly related pairs by some method and generate a decomposition of the superfamily into positions which are important for structural change in close homologues compared to positions important for distantly related proteins.

This is potentially useful for improving the selection of templates for modelling proteins using distant templates however it requires a very accurate alignment between the templates and the target which may be difficult to achieve.

## Conclusion

We have presented the results of applying a simple correlation based method previously used in the identification of functional residues to the question of determining a relative scale of structural importance for residue positions in a set of aligned protein sequences. The results agree well with common knowledge of protein sequence-structure relationships but also provide some novel insights. This method should be useful in future analyses of protein sequence/structure/function relationships.

## Methods

### Data Derivation and Preparation

Six superfamilies of homologous proteins (Table 1) were chosen from the CATH database version 3.0 [13]. The families were chosen to provide a reasonably diverse set with structural pairs distributed as evenly as possible over a wide range of similarity values and cover each of the three structural classes (mostly alpha, mostly beta, alpha and beta).

All structures within a family were first structurally aligned as pairs using the TM-align program [16]. The percentage identity was calculated for each pair from these alignments. To reduce bias in the analysis, we derived the largest non-redundant set of structures (with less than 90% mutual pairwise sequence identity derived from the structural alignments) using a greedy, graph-based method which generates a data set by removing the sequence with the most neighbours of 90% or greater identity at each step until only singletons remain. The sets were manually examined to ensure that structures of low quality were identified and removed if necessary; however this was avoided where possible since this is difficult to automate.

The process for manual vetting of results was as follows: the sequence identity and RMSD values produced by TM-align were plotted for each family. Points lying substantially outside the trend for that superfamily were examined and if it was found that the plot for an individual structure against all other superfamily members had an unusual trend it was removed from the set. Where a small number of examples of a single family were found these were also removed as they would bias the analysis. The multiple alignments of the six superfamilies are provided as an additional file [see additional file 2].

For each non redundant set, we then generated a structure-based multiple sequence alignment using 3D-COFFEE [17], which generates a progressive multiple sequence alignment guided by pairwise structural alignments generated with SAP [18].

### Structural Similarity Scores

Pairwise structural similarity scores were calculated in two ways: the RMSD score returned by TM-align was used directly from the results of the structural alignment. However since this process also optimizes the set of equivalent residues it is sometimes inconsistent where families have large loops. As a second measure we also derived RMSD scores using superpositions based on equivalences taken from the multiple alignments. All columns without gaps were used to calculate these. Initially we also generated RMSD values with SAP [18] however these did not substantially differ from those generated by TM-align [16] and therefore were not used in the analysis.

### Positional Sequence/Structure Correlations

Information about the relationship between positional change and structural change was derived with a modification of the mutational behaviour method of Mesa *et al*. [14] which was previously applied to the determination of functionally important residues.

For each column in the alignment, every residue pair is scored using a substitution matrix (BLOSUM62 and PAM250 were used in this case; 19, 20), all comparisons with gaps scoring zero. The Spearman rank correlation coefficient (*ρ*) is then calculated between the vector of these scores and the vector of corresponding structural similarity scores for the whole proteins. In order to remove the correlation due to the global sequence/structure relationship we converted these to partial correlations using the equation

$$\rho_{pcc}=\frac{\rho_{xy}-(\rho_{xz}\cdot \rho_{yz})}{\sqrt{1-\rho_{xz}^{2}}\cdot \sqrt{1-\rho_{yz}^{2}}}$$

Where ρ_xy_ is the columnwise Spearman correlation with the RMSD score, ρ_xz_ is the columnwise Spearman correlation with sequence similarity scores and ρ_yz_ is the global correlation between RMSD scores and sequence similarity scores.

### Significance of Structural Correlations

Correlation scores were found for each column using the two different RMSD measures and the two score matrices to produce four correlation values. Columns were assigned to one of three categories: structurally informative (+), structurally uninformative (-) or structurally neutral (0). To make the analysis as conservative as possible the columns were assigned to the structurally uninformative category unless all four correlations were found to be significantly correlated or anticorrelated for that column using the T-distribution with N-2 degrees of freedom and calculating T using the approximation

$$T=\frac{\rho }{\sqrt{\frac{\left(1-\rho^{2}\right)}{\left(n-2\right)}}}$$

We used a T-threshold of 1.96 to define a column as non-neutral in the appropriate direction.

### Residue Classifications

Residues were grouped according to three classifications: residue type, secondary structural state and solvent accessibility. Residue types were used directly, as were secondary structural states, which were taken from DSSP [21]. Functional residues were assigned using direct analysis of the structures as previously described in reference [22]. We defined five classes of residue: Functional, Interface, Buried Core, Exposed and Intermediate. Residues were assigned to the Functional class if any sidechain atom was within 4 Å of a non-protein group in the structure, excluding water and sulphates. Relative solvent accessibilities were calculated for non-functional residues using reference values for extended G-G-X-G-G pentapeptides as in reference [22] and assigned to one of four classes: residues which were < 5% accessible in the single domain structure were assigned to the Buried Core class; residues less accessible in the quaternary structure (as defined by PQS, [23]) than the single domain structure were assigned to the Interface class; residues exposing > 25% of their area to solvent in both structures were assigned to the Exposed class. All other residues were assigned to the Intermediate class (following the scheme in ref. [24]).

We assessed the strength of the association between structurally informative residues and predefined classes using chi-square tests. In each case the null hypothesis was of random associations between the class and the three structural types. In each case we applied the Bonferroni correction for multiple hypotheses.

### Subset Similarity Scoring

Similarity scores for subsets of residues were the mean C_valdar _conservation scores [25] over aligned residues in the selected subset, calculated using the BLOSUM62 matrix. This score essentially normalizes substitution matrix scores to the interval [0–1].

## Authors' contributions

DTJ conceived the study, MIS performed experiments and analysed results; both authors designed experiments and wrote the paper.

## Supplementary Material

Additional file 1 **PERL scripts for calculating correlations for MSA columns column_correl_final.pl) and generating data for associations between correlations and structural types (type_ss_stats.pl).** Click here for file

Additional file 2 **Sets of quality values, multiple sequence alignment (aln format) and CC values for each of the six datasets.** Click here for file
